# The weakly electric fish, *Apteronotus albifrons*, actively avoids experimentally induced hypoxia

**DOI:** 10.1007/s00359-021-01470-w

**Published:** 2021-03-10

**Authors:** Stefan Mucha, Lauren J. Chapman, Rüdiger Krahe

**Affiliations:** 1 Institute of Biology, Humboldt-Universität zu Berlin, Unter den Linden 6, 10099 Berlin, Germany; 2 Department of Biology, McGill University, Montreal, QC H3A 1B1 Canada

**Keywords:** Dissolved oxygen, Active sensing, Electric organ discharge, Gymnotiform, Shuttle-box choice chamber

## Abstract

Anthropogenic environmental degradation has led to an increase in the frequency and prevalence of aquatic hypoxia (low dissolved oxygen concentration, DO), which may affect habitat quality for water-breathing fishes. The weakly electric black ghost knifefish, *Apteronotus albifrons*, is typically found in well-oxygenated freshwater habitats in South America. Using a shuttle-box design, we exposed juvenile *A. albifrons* to a stepwise decline in DO from normoxia (> 95% air saturation) to extreme hypoxia (10% air saturation) in one compartment and chronic normoxia in the other. On average, *A. albifrons* actively avoided the hypoxic compartment below 22% air saturation. Hypoxia avoidance was correlated with upregulated swimming activity. Following avoidance, fish regularly ventured back briefly into deep hypoxia. Hypoxia did not affect the frequency of their electric organ discharges. Our results show that *A. albifrons* is able to sense hypoxia at non-lethal levels and uses active avoidance to mitigate its adverse effects.

## Introduction

All water-breathing fishes depend on dissolved oxygen (DO) for their long-term survival (Kramer 1983). In many aquatic ecosystems, DO concentration fluctuates naturally and can reach critically low levels due to water stratification and high temperatures as well as biological decomposition and respiration processes (Kramer 1983; Graham 1990; Diaz 2001), a condition called aquatic hypoxia. Natural hypoxia is particularly widespread in tropical freshwaters where high water temperature elevates organic decomposition and reduces oxygen solubility. In recent years, anthropogenic influences, such as global climate change and eutrophication of water bodies, have led to a global increase of frequency and severity of hypoxic events. This development has been extensively studied for oceanic and coastal regions (Goldberg 1995; Pörtner 2001; Pörtner and Knust 2007; Diaz and Rosenberg 2008; Pörtner and Peck 2010; Schmidtko et al. 2017; Breitburg et al. 2018), and some freshwater lacustrine systems (Jenny et al. 2016a, b). Hypoxia, whether natural or anthropogenic in origin, can affect species composition of ecosystems in a variety of ways that can range from promoting hypoxia-tolerant species (Shoji et al. 2005) to the complete loss of biodiversity as in the so-called dead zones (Diaz and Rosenberg 2008). Many fishes respond to hypoxia by migrating to better oxygenated habitats if available (Pihl et al. 1991; Crampton 1998; Bell and Eggleston 2005; Brown et al. 2015). Avoidance behavior may provide individuals with the flexibility to mitigate hypoxic stress without the immediate need for physiological or biochemical adjustments, though this is largely speculative. Furthermore, not all fish species show active avoidance behavior (Cook et al. 2011), and some hypoxia-tolerant species even actively seek hypoxic zones as refuges from predators (Chapman et al. 2002; Anjos et al. 2008; Vejřík et al. 2016). The ability of fish to venture into hypoxic areas may therefore be important in evaluating costs and benefits of hypoxia avoidance. To broaden our understanding of hypoxia avoidance behavior in fish, we subjected a Neotropical weakly electric fish to oxygen choice experiments.

The black ghost knifefish, *Apteronotus albifrons,* belongs to the gymnotiform weakly electric fishes, a group that constitutes a major food web component in many floodplains and river channels of the Amazon and Orinoco basins (Lundberg et al. 1987; Crampton 1996). Weakly electric fish generate an electric field around their body by discharging a specialized electric organ. *Apteronotus albifrons* produces wave-type electric organ discharges (EODs): continuous, quasi-sinusoidal EODs with individual-specific frequencies between 800 and 1200 Hz (Hopkins 1976; Crampton and Albert 2006). By sensing perturbations of the electric field, weakly electric fish are able to navigate and locate objects, such as prey in dark and turbid waters (Lissmann and Machin 1958) and communicate with conspecifics (Heiligenberg 1989). Their EODs are easy to measure, which makes them particularly well suited to research on the energetics of sensation and communication. It has been suggested that the generation of EODs is an energetically demanding process (Stoddard and Salazar 2011; Salazar et al. 2013; Markham et al. 2016), for which weakly electric fish compensate by reducing the amount of energy that is allocated to other metabolic functions (Stoddard and Salazar 2011). However, dedication of metabolic energy to signal production might impose constraints on the capacity of weakly electric fish to tolerate hypoxia (Markham et al. 2016). In particular, wave-type species that appear to be unable to reduce their EOD frequency under hypoxic stress (Crampton 1998; Reardon et al. 2011) and employ energetically demanding behavior, such as scan-swimming for object detection (Julian et al. 2003), are likely to be subject to these constraints. Accordingly, wave-type gymnotiforms that produce high-frequency EODs have been observed to be restricted to well-oxygenated habitats (Crampton 1998). Our study species, *A. albifrons*, falls within this category and is therefore likely to be hypoxia-sensitive.

The objective of our study was to find out how hypoxia affects the behavior and EOD frequency of *A. albifrons*. We quantified swimming behavior and EOD frequency while exposing fish to progressive hypoxia in a shuttle-box choice chamber and offering a normoxic refuge at all times. We hypothesized that *A. albifrons* will begin to avoid hypoxia at moderate DO levels as part of their natural respiratory strategy. Based on a study of the closely related brown ghost knifefish, *Apteronotus leptorhynchus*, which found a reduction in EOD amplitude but not in EOD frequency under hypoxic stress (Reardon et al. 2011), we hypothesized that *A. albifrons* will not modulate their EOD frequency while experiencing hypoxia. To our knowledge, this is the first study of a weakly electric fish in a behavioral hypoxia avoidance experiment.

## Materials and methods

### Experimental animals and housing conditions

We used farm-bred *Apteronotus albifrons* (Linnaeus, 1766) obtained from a commercial supplier (AQUAlity Tropical Fish Wholesale, Inc., Mississauga, Ontario, Canada). Experiments were performed with 16 individuals with a mean body mass of 3 g (range 1.7–4.2 g), a mean standard body length (SBL) of 8.9 cm (range 7.6–10.4 cm), and an electric organ discharge (EOD) frequency of 807–1151 Hz at 26 °C. Sexually mature *A. albifrons* typically have a SBL of 14–30 cm and a body mass of at least 20 g (Nelson and MacIver 1999; Dunlap and Larkins-Ford 2003; Serrano-Fernández 2003). Thus, it is likely that most, if not all, of our experimental animals were sexually immature, and we did not distinguish fish by sex for data analysis. This was confirmed by gonadal inspection in one case. Fish were housed in tanks of 75 L in groups of 3–4 individuals per tank. Individual fish were separated with plastic mesh tank dividers, and each fish had access to one PVC tube as shelter. The water temperature averaged 25.7 °C (range 25.4–25.9 °C), conductivity 200 µS (190–210 µS), and pH 7.1 (6.8–7.3). Normoxic air saturation levels (> 95%) were maintained by bubbling air into the tanks. Fish were kept at a 12:12 h light:dark photoperiod and were fed daily a small amount of frozen bloodworms (chironomid larvae, Hikari Sales USA, Inc., Hayward, California, USA). Controlled conditions were maintained for a minimum of two weeks before the start of experiments.

### Hypoxia avoidance setup

**Fig. 1:**
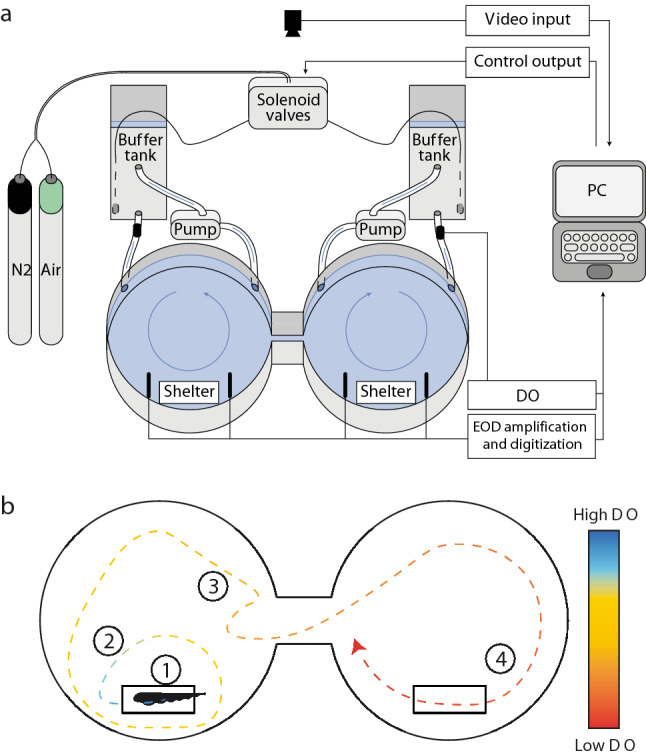
**a** Schematic of shuttle-box oxygen choice chamber. Blue arrows indicate water flow in the compartments and between choice chamber and buffer tanks. Plexiglas lids, heating system and grounding electrode are not shown. **b** Sketch of fish trajectory of hypoxia avoidance behavior, for illustration. 1: Fish remains in shelter on preferred side in high DO; 2: Fish increases locomotor activity during intermediate-low DO but does not change side; 3: Low DO drives fish from preferred side; 4: Fish stays on normoxic side and avoids hypoxic side during deep hypoxia. Line color illustrates DO level

We used a shuttle-box dissolved oxygen choice chamber (Loligo Systems Inc., Denmark) to quantify hypoxia avoidance behavior (Fig. 1). The choice chamber consisted of two circular compartments (each 50 cm in diameter) connected by a central passage (*W* = 8.5 cm, *L* = 14 cm). A PVC tube (*L* = 15 cm, inner diameter 2.6 cm) was placed symmetrically in each compartment as shelter to minimize stress and to reduce arbitrary swimming activity. The two compartments received water from separate buffer tanks where the dissolved oxygen (DO) was controlled by bubbling air or nitrogen gas into the water. DO was measured before the water entered the compartments with a galvanic oxygen probe (MINI-DO, Loligo Systems). Water exchange between the choice chamber and buffer tanks was maintained with aquarium pumps (Universal Pumpe 1048, EHEIM GmbH &Co.KG, Germany). Water temperature was maintained via silicon rubber heating mats (OMEGA Engineering, Inc., USA) that were wrapped around the buffer tanks and controlled by a thermostat (Inkbird Tech C.L., China) with a submerged temperature sensor placed in the passage between the compartments. Circular acrylic glass lids were submerged in the choice chamber ca. 1 cm below the water surface to reduce the diffusion of atmospheric oxygen into the water and to prevent fish from accessing the surface during trials.

Fish position was recorded with a camera (UI-1640-SE-C-GL, IDS Imaging Development Systems GmbH, Germany) mounted above the shuttle-box. ShuttleSoft software (ver. 2.6.4, Loligo Systems) was used to log fish position and DO and to control air saturation in the buffer tanks via a DAQ-M device (Loligo Systems Inc., Viborg, Denmark), which operated solenoid valves at the gas tubing. EODs were measured via submerged carbon rod electrodes in the choice chamber. Two electrodes were placed in each compartment near the PVC tube that served as a shelter, and one grounding electrode was placed in the passage between the compartments. The choice chamber was set up in an isolated room to minimize disturbance.

### Hypoxia avoidance trials

Trials were conducted at water parameters resembling housing conditions (conductivity of 200 µS and pH of 6.9–7.3) with a total water volume of 60 L. Due to slight variation in room temperature, water temperature at the initiation of the trials varied between 25.4 and 25.9 °C. During trials, temperature decreased on average by 0.15 °C (0–0.4 °C) due to room ventilation.

**Fig. 2:**
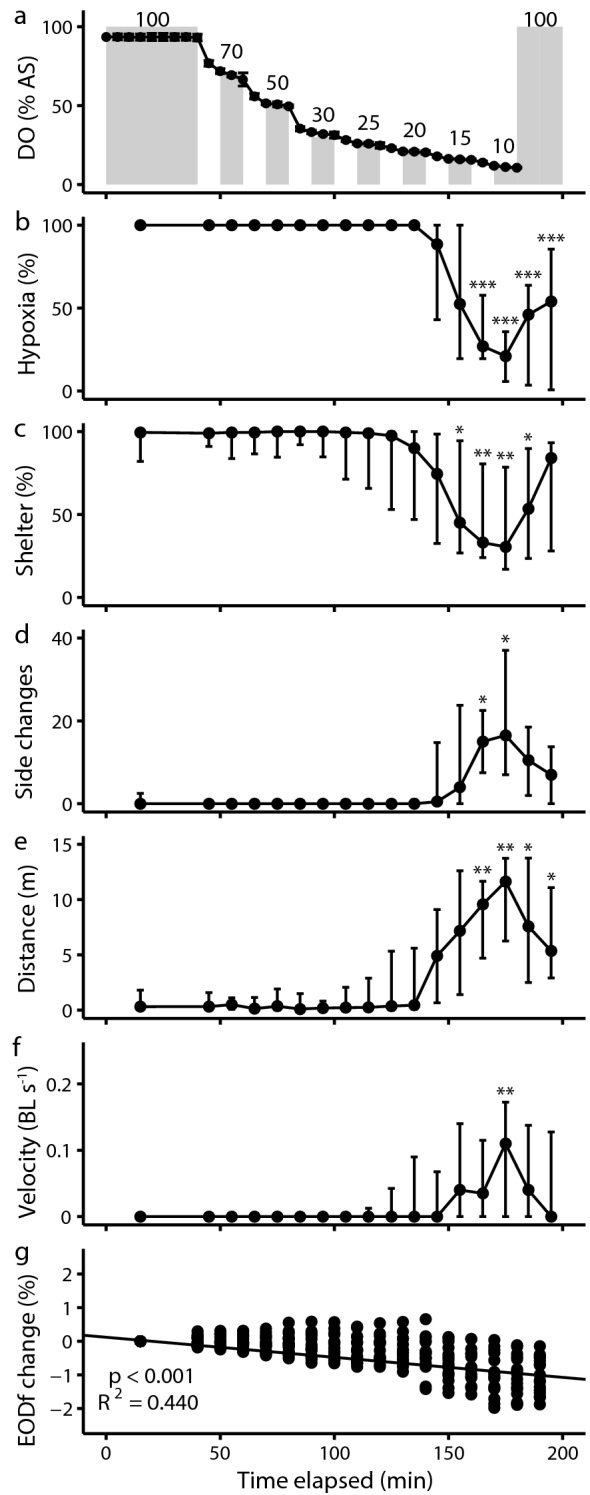
Behavioral responses of *A. albifrons* during hypoxia avoidance trials. **a** DO concentration (% air saturation) during a control trial. Grey bars: target DO concentration, black circles: control measurements. **b** Percentage of time spent in the hypoxic compartment. We induced hypoxia in the compartment where the fish preferred to stay. **c** Percentage of time spent in the tubes that served as shelter. **d** Number of side changes in shuttle box. **e** Distance travelled in the entire choice chamber. **f** Swimming velocity as body lengths per second in the entire choice chamber. **g** Linear regression of EOD frequency change as percentage change from baseline EOD frequency based on LMM. Circles represent median values, vertical bars represent first and third quartile, values from the first 40 min were pooled as normoxic baseline behavior, asterisks indicate statistically significant differences from normoxic baseline (pairwise Wilcoxon rank-sum tests with Holm–Bonferroni correction of *p*-values, *p* < 0.05 *, *p* < 0.01 **, *p* < 0.001 ***)

Fish were fasted for 36 h prior to experiments to ensure a post-absorptive state. For each trial, one fish was introduced into the choice chamber in the afternoon and left for 16 h to acclimate overnight. The side of introduction was chosen randomly, and the fish could freely shuttle between both compartments throughout the experiment. Water was aerated until the start of trials, and measurement devices were calibrated to 100% air saturation before each trial. Trials started in the morning at 9:30 h (30 min after the onset of the light photoperiod). Both compartments were maintained at > 95% air saturation for 40 min to record baseline behavior at normoxia. Each of the 16 fish exhibited a pronounced preference for one of the two compartments during baseline controls. We subsequently induced stepwise hypoxia in the compartment of the choice chamber where the fish preferred to stay while maintaining water in the non-preferred compartment at high DO levels (> 80% air saturation). DO concentration was incrementally lowered to the following air saturation levels: 70%, 50%, 30%, 25%, 20%, 15%, and 10%. Each DO concentration was maintained for 10 min followed by a 10 min decrease to the next lower concentration (Fig. 2a). After the lowest DO concentration was reached, the hypoxic compartment was reoxygenated, and data acquisition was continued for 20 min. The total trial duration (baseline + hypoxia induction + reoxygenation) was 200 min. Upon completion of a trial, the fish was weighed and its SBL measured.

### Data acquisition and processing

During each trial, the fish position was tracked from above based on image contrast. X and Y coordinates, distance moved (cm), swimming velocity (cm s^−1^), and air saturation (%) were logged every second. The log file was processed with Microsoft Excel®2010 (Microsoft Corp., Redmond, Washington, USA) and R (ver. 3.2.5, https://www.r-project.org). Electrical EOD recordings were band-pass filtered (300 Hz–5 kHz) and amplified (1000 × gain, A-M Systems Model 1700, USA). Signals were then digitized with a sample rate of 20 kHz (National Instruments USB-6211, USA) and saved on a computer using custom-written Matlab programs (The MathWorks Inc., USA).

### Statistical analyses

All statistical analyses were performed with R (ver. 4.0.0, https://www.r-project.org). Figures were created using R and captions were edited using Adobe Illustrator (Adobe Systems Software Ltd., Ireland).

#### Side preference during normoxic baseline recordings

Side preference was tested with a two-sided single-sample Wilcoxon rank-sum test on residence time in the preferred compartment against the null hypothesis that fish would spend 50% of the time in each of the compartments (= no preference). As fish did not tend to rest in the passage between compartments, we ignored this possibility for this test.

#### Swimming behavior

Residence time in hypoxia (% of time spent in the hypoxic compartment), time in shelters (% of time spent in PVC tubes), number of side changes, distance moved (meters, m) and average swimming speed (body lengths per second, BL s^−1^) were summarized as medians over the 40 min normoxic baseline period and each following 10 min interval of the trial.

All movement variables were tested for significant changes throughout the trial using Friedman’s rank-sum test with experimental time as independent variable. In case of a significant result, this was followed by pairwise Wilcoxon rank-sum tests with Holm–Bonferroni correction of *p*-values to identify the experimental time at which a significant deviation from normoxic baseline recordings occurred.

Due to water exchange between the choice chamber and buffer tanks, there was a constant circular water current in the compartments. Swimming speed and distance were not corrected for water current; rather, these metrics are used to indicate changes of swimming activity, such as stationary behavior vs. exploration/avoidance.

#### Electric organ discharges

EOD frequency was extracted from recordings using custom-written routines in Matlab R2017a (The MathWorks, Inc., Natick, Massachusetts). Recorded signals were Fourier-transformed, and the frequency with the highest power spectral density estimate (frequency resolution 0.076 Hz) was picked as the EOD frequency for every second of the recording. Median EOD frequency over the 40 min baseline period and for each subsequent 10 min interval of the trial was calculated for each fish. To account for individual differences in the baseline EOD frequency of each fish, values were normalized as percent change from normoxic baseline values for each of the 10 min intervals following baseline recordings. To test for an effect of hypoxia on EOD frequency, we used a random-slope linear mixed-effect model (LMM) with change of median EOD frequency as dependent variable. Based on AIC scores, the best fit was achieved by including the interaction of inversed DO concentration with residence time in hypoxia (i.e. the lower the DO concentration in which the fish stayed, the higher the interaction term). Experimental time was treated as a fixed effect and fish ID as random effect. The intercept of the LMM was set to zero.

EOD amplitude was strongly affected by the position and orientation of the fish relative to the recording electrodes. As we could not always determine the exact fish position and orientation (e.g. when fish were in their shelters or swimming in the passage between compartments), we excluded EOD amplitude from our analysis.

#### Hypoxia avoidance threshold

We quantified the threshold for hypoxia avoidance by modelling the correlation between residence time in hypoxia and DO concentration with a modified version of the program for *P*_crit_-determination by Yeager and Ultsch (1989). The program estimates the best fit of two linear regressions to a dataset, iteratively minimizing their residual sum of squares. Two LMMs with random intercepts were calculated with residence time in the hypoxic compartment as the dependent variable. DO concentration was included as fixed effect, and fish ID was included as a random effect. The hypoxia avoidance threshold was defined as the DO concentration at which the regression lines of both LMMs intersected. Conditional and marginal *R*^2^ values of both LMMs were calculated based on the method by Nakagawa and colleagues (Nakagawa et al. 2013). *T*-test statistics and *p*-values for the null hypothesis of zero correlation between residence time in hypoxia and DO were calculated with degrees of freedom obtained through Satterthwaite approximation. The R code for these procedures was adapted from the rMR package (Moulton 2018).

### Repeatability trials

To test the repeatability of our experimental protocol and results, hypoxia avoidance trials were repeated after 4 weeks with five fish. Trials were conducted as described above, and residence time in the hypoxic compartment was tested for differences between the first trial and the repeatability trial using a two-way repeated measures ANOVA with DO concentration as between-subject effect, and experimental day as within-subject effect.

## Results

### *Apteronotus albifrons* shows pronounced side preference and stationary behavior at normoxia

During normoxic baseline recordings, all 16 individuals showed a pronounced preference for one compartment of the shuttle-box choice chamber over the other (Wilcoxon single-sample rank-sum test, *p* < 0.001) with 10 fish spending the whole baseline period exclusively on one side and no fish spending less than 79% of the time on one side. Among all 16 fish, the two compartments were chosen 8 times each as preferred side, indicating that there was no bias to either side of the choice chamber. Further, there was no correlation between the side of introduction and the preferred side; 7 fish chose the side of introduction and 9 fish settled into the other side of the choice chamber. During this period, fish predominantly rested in the PVC tubes that were provided as shelters. We exploited this side preference as the compartment in which to lower the dissolved oxygen concentration.

### *Apteronotus albifrons *upregulates swimming activity and reduces shelter preference in deep hypoxia

Moderate hypoxia above 20% air saturation did not affect fish behavior. Fish predominantly rested in their shelters and showed no significant deviations from normoxic baseline behavior. Below 20% air saturation, swimming activity increased and fish spent less time in the hypoxic compartment and more time outside of their shelters (Fig. 2). After leaving the hypoxic compartment, fish remained active and occasionally ventured back to the hypoxic side. This sustained change from stationary swimming to active roaming was evident in significantly reduced shelter-seeking behavior below 20% air saturation (Wilcoxon rank-sum test with Holm *post hoc* correction, *p* < 0.05, Fig. 2c) and a significant increase in the number of side changes and distance travelled at air saturations below 15% (Wilcoxon rank-sum test with Holm *post hoc* correction, *p* < 0.05, Fig. 2d, e).

At the lowest DO concentration of 10% air saturation, fish showed the strongest deviations from baseline behavior. During this 10-min interval, they spent the least amount of time in the hypoxic compartment (median: 21%, interquartile range 6–36%) and in a shelter (30%, IQR: 17–79%), shuttled most often between sides (16.5 side changes, IQR: 7–37 side changes), and travelled the longest distance (11.64 m, IQR: 6.25–13.74 m) with the highest median velocity (0.11 BL s^−1^, IQR: 0–0.17 BL s^−1^; Fig. 2f). Comparing both compartments, we found that fish significantly increased the time in shelter, travelled distance, and residence time in the normoxic compartment during this most active phase of the trial (pairwise Wilcoxon rank-sum test, *p* < 0.05).

After the hypoxic compartment was reoxygenated, fish showed no clear side preference (pairwise Wilcoxon rank-sum test with Holm *post hoc* correction, *p* < 0.001). Swimming activity and distance travelled decreased and approached baseline values (Wilcoxon rank-sum test with Holm post hoc correction, velocity *p* > 0.05), and fish returned to spending the majority of the time in a shelter. Residence times in the hypoxic compartment did not differ significantly between repeatability trials and original trials (ANOVA: *F* = 0.114, *p* = 0.753).

### *Apteronotus albifrons *does not modulate electric organ discharge frequency in hypoxia

Median EOD frequency decreased marginally throughout hypoxia avoidance trials, but did not show a clear correlation with DO concentration as the largest change of − 1.08% from normoxic baseline EOD frequency occurred at the end of the trial and not at the lowest air saturation (median value across all fish, Fig. 2g). According to LMM estimates, this decrease resulted from a small but significant negative effect of experimental time on frequency. The most likely cause of the marginal decrease of median EOD frequency is the slight cooling of water temperature during trials, which amounted to an average decrease of 0.15 °C. Assuming a *Q*_10_ value of 1.55 for EOD frequency (Dunlap and Ragazzi 2015), temperature change explains a reduction of EOD frequency by 0.7%. The interaction term of residence time in hypoxia and inverted DO concentration had a negligible positive effect on frequency, indicating that fish did not reduce their EOD frequency in response to hypoxia.

### Linear mixed-effect models suggest hypoxia avoidance threshold at 22% air saturation

**Fig. 3:**
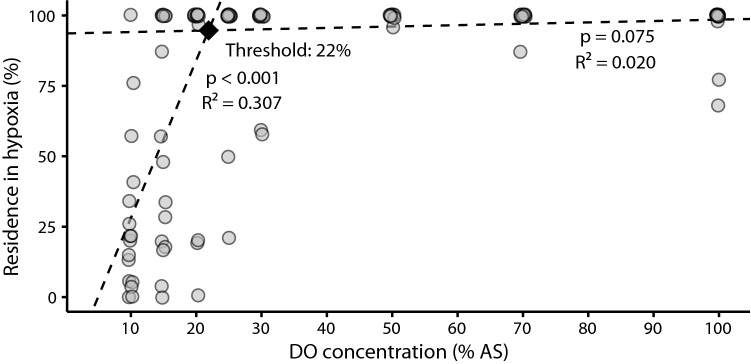
Residence time in the hypoxic compartment as function of the dissolved oxygen in % air saturation. Grey circles represent the percentage of time that individual fish spent in the hypoxic compartment at each air saturation that was established in this compartment (*n* = 16 fish, points are jittered along the *x*-axis to reveal overlapping measurements), dashed lines represent linear regressions based on LMMs, the black diamond at their intersection indicates the computed hypoxia avoidance threshold, *R*^2^ = adjusted marginal *R*^2^, *p* = probability of zero correlation between air saturation and residence in hypoxia

To determine the threshold for the onset of hypoxia avoidance, we modelled the impact of hypoxia on residence time in the hypoxic compartment using two LMMs with random intercepts and slopes (Table 1, Fig. 3). Based on this method, DO concentration above and including 25% air saturation had no effect on the residence time in the hypoxic compartment (adjusted marginal *R*^2^ = 0.02, *p* = 0.075, Table 1). However, below 25% air saturation, DO concentration significantly affected the residence in the hypoxic compartment (adjusted marginal *R*^2^ = 0.307, *p* < 0.001, Table 1) as fish began to increasingly avoid the hypoxic compartment. Inter-individual variation of the hypoxia avoidance response was high with 13 fish spending less than 50% of the time in the hypoxic compartment and one fish showing no hypoxia avoidance response at the lowest DO concentration of 10% air saturation. We computed the intersection of both linear regressions at 22% air saturation as the average threshold for the onset of hypoxia avoidance behavior.

**Table 1:** Linear mixed-effect model (LMM) estimates for the change of residence time in hypoxia (n = 16 fish)

Fixed Effects	Estimate	SE	*DF*	*t*	*p*	*R*^2^
Pre-threshold						
Intercept	93.639	2.437	82.544	38.425	< 0.001	
DO	0.049	0.027	109.462	1.797	0.075	0.02
Post-threshold						
Intercept	− 27.938	14.605	44.489	− 1.913	0.062	
Air saturation	5.6	0.849	31	6.596	< 0.001	0.307

The data were split into two subsets with one LMM each by iteratively minimizing the residual sum of squares. The results are two LMMs, one that fits the data while residence time in hypoxia is relatively unaffected by DO (pre-threshold) and one that fits the data from the point when residence time in hypoxia is affected by DO (post-threshold)

*SE* = standard error, *DF* = degrees of freedom, *t* = test statistic for null hypothesis of zero correlation, *R*^2^ = adjusted marginal *R*^2^ = for LMM fit

## Discussion

### Increased locomotor activity drives hypoxia avoidance at safe oxygen levels

Our study shows that *Apteronotus albifrons* use an active avoidance strategy to escape hypoxia. Hypoxia avoidance behavior followed a threshold dynamic with little or no effect of moderate hypoxia and a strong effect of deep hypoxia on swimming behavior. During hypoxia avoidance, fish showed increased locomotor activity, reduced shelter-seeking behavior, and decreased residence time in hypoxia with a high inter-individual variation. We found no clear effect of hypoxia exposure on EOD frequency. We identified the threshold for the onset of hypoxia avoidance at approximately 22% air saturation. This value lies above the threshold for aquatic surface respiration for *A. albifrons* (ASR_50_, the oxygen level at which fish spend 50% of their time engaged in breathing water from the surface film, Lewis 1970), estimated as 18.3% (Vassileva, Krahe, and Chapman, unpublished). The threshold for hypoxia avoidance in *A. albifrons* also falls well above the critical oxygen tension (*P*_crit_) for the closely related species *Apteronotus leptorhynchu*s and a distantly related gymnotiform, *Eigenmannia virescens*. *P*_crit_, the oxygen partial pressure below which the oxygen consumption rate of the fish switches from oxygen regulation to oxygen conformation, was estimated as 10.5% air saturation for *A. leptorhynchus* and 7.1% for *E. virescens* (Reardon et al. 2011). We suggest that the onset of an avoidance reaction at 22% air saturation provides *A. albifrons* with the flexibility to seek better oxygenated areas before hypoxic stress impairs its physiology. This is consistent with anecdotal observations that wave-type gymnotiform fishes are typically found in habitats with a DO concentration of 40–60% air saturation and avoid swimming into hypoxic to anoxic waters (Crampton 1998). To establish a clearer picture of how behavioral and physiological thresholds interrelate, the determination of the *P*_crit_ of *A. albifrons* and a repetition of hypoxia avoidance trials using these closely related species are warranted.

Hypoxia avoidance co-occurred with a sustained upregulation of locomotor activity and decrease of shelter-seeking behavior. At first glance, this effect seems counterintuitive as a short bout of activity would have sufficed to leave the hypoxic compartment of the choice chamber while minimizing energy expenditure and risk due to time spent outside the shelter. The lasting upregulation of locomotor activity could be an effect of the initial displacement from their shelter. In a natural setting, hypoxic areas are likely to be more extended than in our setup and a sustained increase of locomotor activity might be necessary to reach better oxygenated areas. The active response to hypoxia described here is similar to the behavioral responses of other species, such as red hake (*Urophycis chuss,* Bejda et al. 1987), tuna (*Katsuwonus pelamis* and *Thunnus albacares,* Bushnell and Brill 1991), Atlantic herring (*Clupea harengus*, Domenici et al. 2000), weakfish (*Cynoscion regalis*, Brady et al. 2009), and rainbow trout (*Oncorhynchus mykiss,* Poulsen et al. 2011). Overall, these cases support the notion that active hypoxia avoidance strategies are employed by fish species with an active lifestyle.

We used juvenile *A. albifrons* to quantify hypoxia avoidance behavior in this study, with a small range in body length of 7.6–10.4 cm. It is conceivable that adult fish would have shown a different response to hypoxia. Relationships between body size and metrics of hypoxia tolerance vary among studies and may reflect selective pressures associated with differences in habitat use or life-style among life stages of fishes (Nilsson and Ostlund-Nilsson 2008; Chapman 2015). However, Nilsson and Ostlund-Nilsson (2008) concluded that body size per se in fish should have little effect on the oxygen uptake capacity under hypoxia as the respiratory surface area and metabolic rate scale similarly over a wide range of body sizes (Nilsson and Ostlund-Nilsson 2008). Although larger individuals tend to have a higher capacity for anaerobic metabolism than smaller conspecifics (Nilsson and Ostlund-Nilsson 2008), our results suggest that *A. albifrons* avoid hypoxia to prevent anaerobic physiological conditions. Therefore, we would expect any size-dependent effects on hypoxia avoidance behavior to be small. However, it is possible that sexual dimorphism in other traits, such as EOD production (Dunlap et al. 1998; Dunlap and Larkins-Ford 2003), could drive different hypoxia avoidance responses in adult fish.

### Electric organ discharge frequency is not a part of the hypoxia mitigation strategy of *Apteronotus albifrons*

We found a small decrease of median EOD frequency throughout trials that was most likely caused by slight cooling of the tank water during experiments. The neuronal pacemakers underlying the generation of EODs in wave-type weakly electric fish are the most stable biological oscillators known (Moortgart et al. 1998, 2000); however, discharge frequency is tightly coupled to ambient temperature (Dunlap et al. 2000; Dunlap and Ragazzi 2015), making EOD frequency a highly sensitive indicator of temperature change. Given the small average temperature decline of 0.15 °C, we expect its effect to be noticeable as a slight change in EOD frequency, but to be negligible with respect to other behavioral parameters.

In theory, a reduction of discharge frequency should reduce the energetic cost of the electric sense and thus could be a useful means to survive hypoxia (Salazar et al. 2013). However, there is no evidence that wave-type gymnotiforms employ frequency reduction as a means of saving energy. In contrast, it has been proposed that wave-type gymnotiforms are incapable of effectively reducing their EOD frequency under hypoxic stress (Crampton 1998; Markham et al. 2009; Reardon et al. 2011). As the fish in our study were not forced to remain under hypoxic conditions, we can make no inferences about their capacity to regulate EOD frequency under hypoxic stress. Instead, our results suggest that *A. albifrons* leave hypoxia before EOD frequency is affected, regardless of whether by active regulation or as a mere consequence of hypoxic stress. This suggests that, even if possible, active regulation of EOD frequency is only relevant under inescapable hypoxic conditions, which may be rare events in the natural habitat of many gymnotiforms.

Another parameter that is relevant for the energetic cost of EODs is their amplitude (Markham et al. 2009; Stoddard and Salazar 2011; Salazar et al. 2013). Because of the changing distance and orientation of the fish relative to the recording electrodes, we were not able to reliably measure the EOD amplitude of freely swimming fish in our study. So far, reduction of EOD amplitude has only been found under inescapable hypoxic conditions approaching the respective *P*_crit_ in *A. leptorhynchus* and *E. virescens* (Reardon et al. 2011). The comparably high hypoxia avoidance threshold of 22% air saturation suggests that *A. albifrons* avoided hypoxia before its EOD amplitude was affected. However, additional experiments are needed to clarify whether the DO concentrations at which fish began to leave hypoxia in our experiment have an effect on EOD amplitude.

### Excursions into hypoxia and inter-individual variation demonstrate flexibility of hypoxia avoidance behavior

Even at 10% air saturation, fish voluntarily ventured into hypoxia for brief excursions. This behavior was somewhat unexpected given that there was no obvious incentive for fish to leave the normoxic compartment. Similar behavior has been found in other fish species, sometimes associated with foraging behavior (Jones 1952; Rahel and Nutzman 1994; Claireaux et al. 1995; Wannamaker and Rice 2000; Herbert et al. 2011; Cook and Herbert 2012). Further, we observed a high inter-individual variation in hypoxia avoidance behavior with residency in the hypoxic compartment varying from 0 to 100% at the lowest DO concentration of 10% air saturation. It has been hypothesized that hypoxia avoidance behavior is not directly triggered by external DO concentration but rather relies on various physiological cues that imply “respiratory distress” (Jones 1952; Cook et al. 2011). This indirect relationship between external DO concentrations and behavioral response allows for the integration of many cues into an avoidance response, thus making it more adaptable to different environmental contexts. Whereas ultimately, the physiological need for oxygen is the driver of hypoxia avoidance, the onset of this behavior could be dependent on the interaction of several relevant factors, such as habitat cover (Hill 1968), presence of predators (Wolf and Kramer 1987), availability and quality of an oxygen refuge (Herbert et al. 2011), and acclimation to different oxygen regimes (Cook et al. 2013). Further, it is possible that other factors, such as behavioral traits (e.g. risk-taking and escape behaviors, Castanheira et al. 2013) or stress-coping styles (Laursen et al. 2011), differentially affect hypoxia avoidance behavior. As *A. albifrons* are likely to experience hypoxia occasionally in their natural habitat, the ability to venture into hypoxic waters without immediate avoidance would allow them to forage or migrate and thus could provide an important fitness benefit. More research on this topic is needed to gain a better understanding of the flexibility of behavioral hypoxia tolerance and the physiological cues that trigger it.

### Conclusion and outlook

We show here that *A. albifrons* use an active hypoxia avoidance strategy that is comparable to that of other fishes with active life styles. Our results suggest that active avoidance serves to mitigate negative implications of hypoxia on sensing and physiology rather than adapting to it. These results are in line with previous studies and field observations of wave-type gymnotiforms (Crampton 1998; Reardon et al. 2011) and suggest a low tolerance of *A. albifrons* to hypoxia below 20% air saturation. With regard to the expected increased prevalence of hypoxia in the future, this proactive avoidance strategy is likely to cause habitat shifts and a reduced abundance of *A. albifrons* in affected habitats. Behavioral trade-offs, such as reduced shelter-seeking behavior and increased energy cost of locomotor functions, could increase the vulnerability of *A. albifrons* to predators and other adverse environmental influences. More hypoxia-related behavioral studies are needed for us to better understand the flexibility of behavior in different environmental contexts and the relationship between physiological and behavioral hypoxia tolerance.

## Data Availability

Raw data from video tracking and processed datasets used for statistical analyses are available online (﻿10.6084/m9.figshare.12280778.v1).

## Abbreviations

AS: Air saturation
BL s^−^^1^: Body lengths per second
DF: Degrees of freedom
DO: Dissolved oxygen
EOD: Electric organ discharge
IQR: Interquartile range
LMM: Linear mixed-effect model
SBL: Standard body length
SE: Standard error

## Code availability

Custom-written R code used for statistical analyses, creation of figures and data processing are available online (﻿10.6084/m9.figshare.12280778.v1).
